# Control Work

**Published:** 1898-05

**Authors:** 


### CONTROL WORK.

*Indiana.* Dr. T. B. Pote, food-inspector of Terre Haute, in the report of the city board of health, receives the warm praise of the board for the very careful manner in which he has performed his duties. Some 19,523 cattle, hogs, sheep, and calves killed for food purposes were inspected. Sixteen cattle and four calves were condemned, and 1369 pounds of meat were condemned. Sixty-five visits were made to dairy farms for inspection purposes. One hundred and seven samples of milk were taken and tested. Preservalin was found in these samples ; some twenty-six dairy animals were condemned for tuberculosis. Practically, all the slaughtering of animals for food is done at one abattoir, and this affords more ready inspection and better control of meat-products in their preparation and sale.

*Missouri.* Benton County has three farms under State quarantine. The horses, three of which have died, are suffering with mange, or Texas itch, supposed to have been introduced through a pony purchased at Kansas City.

*Minnesota.* The veterinary department of the State Board of Health, under Dr. Reynolds, is coöperating with the Bureau of Animal Industry in a way that is proving quite satisfactory. Government inspectors are given authority as representatives of the State Board of Health. In this way they have authority not only over interstate and export traffic, but also with matters that have to do with the State traffic. Parties dealing in diseased animals or handling carcasses unfit for food-purposes cannot escape by claiming that they are intended for sale within the State, or that they are offered only for interstate trade or export. Dr. Reynolds advises other State Boards and State veterinarians, who have not tried this plan, to do so.

